# Supplementary material for: Modeling glioblastoma heterogeneity as a dynamic network of cell states
Source: Mol Syst Biol. 2021 Sep 16;17(9):e10105. doi: 10.15252/msb.202010105 (PMC8444284; doi:10.15252/msb.202010105)
Supplement: Supplementary file 6 — Source Data for Figure 5 [file MSB-17-e10105-s004.zip › Figure5A_sourcedata/GSEA_3071/nosigdb_stateII.GseaPreranked.1622795151171/gsea_report_for_na_pos_1622795151171.html]

Report for na\_pos 1622795151171 [GSEA]

| GS  follow link to MSigDB | GS DETAILS | SIZE | ES | NES | NOM p-val | FDR q-val | FWER p-val | RANK AT MAX | LEADING EDGE || 1 | DOROSZKO\_U3054MG\_CLUSTER\_4 | Details ... | 490 | 0.71 | 5.46 | 0.000 | 0.000 | 0.000 | 979 | tags=78%, list=24%, signal=91% |
| 2 | DOROSZKO\_U3180MG\_CLUSTER\_3 | Details ... | 390 | 0.72 | 5.44 | 0.000 | 0.000 | 0.000 | 696 | tags=69%, list=17%, signal=75% |
| 3 | DOROSZKO\_U3013MG\_CLUSTER\_2 | Details ... | 170 | 0.78 | 5.17 | 0.000 | 0.000 | 0.000 | 719 | tags=85%, list=18%, signal=98% |
| 4 | LARSSON\_STATE\_4 | Details ... | 89 | 0.83 | 4.93 | 0.000 | 0.000 | 0.000 | 579 | tags=89%, list=14%, signal=101% |
| 5 | GAROFANO\_IAVARONE\_PPR\_SUBTYPE | Details ... | 312 | 0.64 | 4.78 | 0.000 | 0.000 | 0.000 | 741 | tags=63%, list=18%, signal=71% |
| 6 | NB\_KI\_CLUSTER5 | Details ... | 75 | 0.80 | 4.59 | 0.000 | 0.000 | 0.000 | 510 | tags=83%, list=12%, signal=93% |
| 7 | SEURAT\_CELLCYCLE | Details ... | 88 | 0.79 | 4.55 | 0.000 | 0.000 | 0.000 | 750 | tags=90%, list=18%, signal=108% |
| 8 | RICHARDS\_NOWAKOWSKI\_SCIENCE\_2017\_MGE\_DIV\_UPREG | Details ... | 470 | 0.59 | 4.51 | 0.000 | 0.000 | 0.000 | 734 | tags=58%, list=18%, signal=62% |
| 9 | RICHARDS\_ZHONG\_NATURE\_2018\_NPCS\_UPREG | Details ... | 112 | 0.70 | 4.35 | 0.000 | 0.000 | 0.000 | 671 | tags=75%, list=16%, signal=87% |
| 10 | FELDMAN\_2020\_S-G2 | Details ... | 290 | 0.59 | 4.27 | 0.000 | 0.000 | 0.000 | 682 | tags=65%, list=17%, signal=72% |
| 11 | FELDMAN\_2020\_S | Details ... | 136 | 0.66 | 4.25 | 0.000 | 0.000 | 0.000 | 639 | tags=71%, list=16%, signal=82% |
| 12 | RICHARDS\_NOWAKOWSKI\_SCIENCE\_2017\_MGE\_IPC2\_UPREG | Details ... | 391 | 0.56 | 4.24 | 0.000 | 0.000 | 0.000 | 724 | tags=58%, list=18%, signal=64% |
| 13 | FELDMAN\_2020\_G1 | Details ... | 128 | 0.67 | 4.23 | 0.000 | 0.000 | 0.000 | 671 | tags=75%, list=16%, signal=87% |
| 14 | SUVA\_G1S\_PMID\_31327527 | Details ... | 27 | 0.89 | 3.91 | 0.000 | 0.000 | 0.000 | 178 | tags=85%, list=4%, signal=88% |
| 15 | RICHARDS\_NEFTEL\_CELL\_2019\_G1\_S | Details ... | 27 | 0.89 | 3.76 | 0.000 | 0.000 | 0.000 | 178 | tags=85%, list=4%, signal=88% |
| 16 | RICHARDS\_NOWAKOWSKI\_SCIENCE\_2017\_IPC\_DIV1\_UPREG | Details ... | 460 | 0.48 | 3.65 | 0.000 | 0.000 | 0.000 | 736 | tags=48%, list=18%, signal=52% |
| 17 | RICHARDS\_NOWAKOWSKI\_SCIENCE\_2017\_MGE\_IPC1\_UPREG | Details ... | 389 | 0.48 | 3.63 | 0.000 | 0.000 | 0.000 | 777 | tags=49%, list=19%, signal=55% |
| 18 | FELDMAN\_2020\_G2-M | Details ... | 208 | 0.48 | 3.32 | 0.000 | 0.000 | 0.000 | 680 | tags=58%, list=17%, signal=66% |
| 19 | DALMO\_BARCODENUMBER\_UNPUB | Details ... | 146 | 0.50 | 3.30 | 0.000 | 0.000 | 0.000 | 446 | tags=47%, list=11%, signal=51% |
| 20 | RICHARDS\_NEFTEL\_CELL\_2019\_G2\_M | Details ... | 39 | 0.68 | 3.28 | 0.000 | 0.000 | 0.000 | 736 | tags=74%, list=18%, signal=90% |
| 21 | SUVA\_G2M\_PMID\_31327527 |  | 39 | 0.68 | 3.26 | 0.000 | 0.000 | 0.000 | 736 | tags=74%, list=18%, signal=90% |
| 22 | RICHARDS\_NOWAKOWSKI\_SCIENCE\_2017\_RG\_EARLY\_UPREG |  | 248 | 0.45 | 3.23 | 0.000 | 0.000 | 0.000 | 654 | tags=44%, list=16%, signal=49% |
| 23 | RICHARDS\_NOWAKOWSKI\_SCIENCE\_2017\_MGE\_RG2\_UPREG |  | 288 | 0.42 | 3.04 | 0.000 | 0.000 | 0.000 | 685 | tags=44%, list=17%, signal=50% |
| 24 | FELDMAN\_2020\_LATEG1 |  | 72 | 0.51 | 2.81 | 0.000 | 0.000 | 0.000 | 666 | tags=64%, list=16%, signal=75% |
| 25 | NB\_KI\_CLUSTER3 |  | 72 | 0.45 | 2.53 | 0.000 | 0.000 | 0.000 | 1389 | tags=69%, list=34%, signal=103% |
| 26 | FELDMAN\_2020\_NEURAL G0 |  | 294 | 0.32 | 2.29 | 0.000 | 0.000 | 0.001 | 681 | tags=40%, list=17%, signal=45% |
| 27 | IVY\_CT |  | 127 | 0.36 | 2.29 | 0.000 | 0.000 | 0.001 | 284 | tags=37%, list=7%, signal=39% |
| 28 | RICHARDS\_CAHOY\_JNEUROSCI\_2010\_ASTRO\_YOUNG |  | 412 | 0.30 | 2.28 | 0.000 | 0.000 | 0.001 | 679 | tags=33%, list=17%, signal=36% |
| 29 | DOROSZKO\_U3054MG\_CLUSTER\_6 |  | 64 | 0.39 | 2.14 | 0.000 | 0.000 | 0.012 | 1334 | tags=61%, list=33%, signal=89% |
| 30 | GAROFANO\_IAVARONE\_LIST\_OF\_NEURAL\_PROGENITOR\_MARKER\_GENES |  | 76 | 0.38 | 2.13 | 0.000 | 0.000 | 0.013 | 420 | tags=42%, list=10%, signal=46% |
| 31 | PARADA\_T1\_UP\_26461091 |  | 45 | 0.43 | 2.10 | 0.000 | 0.001 | 0.024 | 619 | tags=53%, list=15%, signal=62% |
| 32 | WANG\_MESENCHYMAL\_PMID\_28697342 |  | 20 | 0.50 | 1.95 | 0.002 | 0.002 | 0.089 | 1398 | tags=85%, list=34%, signal=128% |
| 33 | DALMO\_EXTINCTION\_UNPUB |  | 20 | 0.50 | 1.93 | 0.000 | 0.002 | 0.104 | 1398 | tags=85%, list=34%, signal=128% |
| 34 | DOROSZKO\_U3180MG\_CLUSTER\_4 |  | 80 | 0.32 | 1.84 | 0.000 | 0.005 | 0.197 | 766 | tags=40%, list=19%, signal=48% |
| 35 | NB\_KI\_CLUSTER7 |  | 97 | 0.29 | 1.76 | 0.003 | 0.008 | 0.318 | 1748 | tags=67%, list=43%, signal=114% |
| 36 | IVY\_CTMVP |  | 53 | 0.32 | 1.70 | 0.009 | 0.013 | 0.466 | 666 | tags=40%, list=16%, signal=47% |
| 37 | ROSEN\_KRONA\_TI\_UNPUBL |  | 34 | 0.35 | 1.65 | 0.022 | 0.019 | 0.591 | 1149 | tags=53%, list=28%, signal=73% |
| 38 | PARADA\_T2\_UP\_26461091 |  | 50 | 0.31 | 1.58 | 0.033 | 0.029 | 0.776 | 619 | tags=42%, list=15%, signal=49% |
| 39 | ANASTASSIOU\_MULTICANCER\_INVASIVENESS\_PMID\_22208948 |  | 26 | 0.38 | 1.57 | 0.046 | 0.029 | 0.786 | 1163 | tags=62%, list=28%, signal=85% |
| 40 | ALMSTEDT\_17Q\_MSIGDB |  | 137 | 0.23 | 1.49 | 0.018 | 0.047 | 0.921 | 1093 | tags=38%, list=27%, signal=50% |
| 41 | RICHARDS\_VERHAAK\_CANCERCELL\_2010\_MESENCHYMAL |  | 63 | 0.27 | 1.49 | 0.032 | 0.047 | 0.929 | 1448 | tags=60%, list=35%, signal=92% |
| 42 | SUVA\_MES1\_PMID\_31327527 |  | 24 | 0.34 | 1.43 | 0.080 | 0.064 | 0.975 | 393 | tags=33%, list=10%, signal=37% |
| 43 | RICHARDS\_NEFTEL\_CELL\_2019\_MES1 |  | 24 | 0.34 | 1.43 | 0.096 | 0.063 | 0.976 | 393 | tags=33%, list=10%, signal=37% |
| 44 | NB\_KI\_CLUSTER24 |  | 54 | 0.27 | 1.41 | 0.074 | 0.069 | 0.985 | 1454 | tags=52%, list=35%, signal=79% |
| 45 | NB\_KI\_CLUSTER18 |  | 50 | 0.27 | 1.40 | 0.093 | 0.069 | 0.985 | 401 | tags=30%, list=10%, signal=33% |
| 46 | NB\_KI\_CLUSTER16 |  | 57 | 0.26 | 1.36 | 0.080 | 0.085 | 0.996 | 816 | tags=44%, list=20%, signal=54% |
| 47 | NB\_KI\_CLUSTER12 |  | 30 | 0.27 | 1.21 | 0.220 | 0.189 | 1.000 | 1636 | tags=67%, list=40%, signal=110% |
| 48 | SINGH\_SOX2\_DOWNREGULATED\_TRANSCRIPTS\_PMID\_28122245 |  | 50 | 0.24 | 1.20 | 0.232 | 0.199 | 1.000 | 892 | tags=34%, list=22%, signal=43% |
| 49 | GANDAL\_AUTISM\_UP\_PMID29439242 |  | 51 | 0.23 | 1.16 | 0.284 | 0.235 | 1.000 | 992 | tags=41%, list=24%, signal=54% |
| 50 | RICHARDS\_NOWAKOWSKI\_SCIENCE\_2017\_U3\_UPREG |  | 337 | 0.13 | 0.94 | 0.672 | 0.593 | 1.000 | 630 | tags=19%, list=15%, signal=21% |
| 51 | KRONA\_ROSEN\_PERIVASCULAR\_INVASION |  | 34 | 0.16 | 0.73 | 0.826 | 0.883 | 1.000 | 1240 | tags=41%, list=30%, signal=59% |
Table: Gene sets enriched in phenotype **na **[plain text format]****
